# Apparent metabolizable energy concentration and ileal amino acid digestibility in cereal grains fed to broiler chickens

**DOI:** 10.5713/ab.24.0398

**Published:** 2024-08-26

**Authors:** June Hyeok Yoon, Changsu Kong

**Affiliations:** 1 Research Institute for Innovative Animal Science, Kyungpook National University, Sangju 37224, Korea; 2 Department of Animal Science, Kyungpook National University, Sangju 37224, Korea; 3 Department of Animal Science and Biotechnology, Kyungpook National University, Sangju 37224, Korea

**Keywords:** Amino Acid, Broiler, Cereal Grain, Digestibility, Metabolizable Energy

## Abstract

**Objective:**

This study aimed to determine apparent metabolizable energy concentrations and ileal amino acid (AA) digestibility in cereal grains and to compare those metabolizable energy values between the total collection and index methods for 21-day-old broilers.

**Methods:**

On day 17 post-hatch, a total of 336 Ross 308 male broilers were assigned to 6 dietary treatments with 8 replicate cages (7 birds/cage). Five experimental diets were formulated to incorporate non-extruded corn, extruded corn, wheat, wheat flour, and barley as the sole source of AA and energy.

**Results:**

Retention of dry matter and nitrogen, and energy concentrations in cereal grains determined by the total collection method were greater (p<0.05) than those determined by the index method. Energy concentrations of non-extruded and extruded corn were greater (p<0.05) than those of wheat, wheat flour, and barley. Wheat flour exhibited greater (p<0.05) ileal AA digestibility than non-extruded and extruded corn. Extruded corn and wheat showed comparable ileal AA digestibility values, whereas barley had the lowest among cereal grains.

**Conclusion:**

Energy concentrations of cereal grains determined by the total collection method were greater than those determined by the index method. Energy concentrations of non-extruded and extruded corn were greater compared to wheat, wheat flour, and barley, irrespective of the method used. The ileal AA digestibility in wheat flour was the greatest, followed by non-extruded corn, extruded corn, wheat, and barley in broilers.

## INTRODUCTION

Cereal grains serve as a major dietary source of energy and carbohydrates, with up to 40% of the annual cereal grain production utilized for animal feed [1]. Global annual yields of corn and wheat have surged to 1,148 and 766 million metric tons, respectively, followed by rice and barley at 755 and 159 million metric tons, respectively [1]. However, the nutrient availability of cereal grains exhibits significant variability, owing to factors such as cultivars, harvest timing, and growing and processing conditions [2]. Understanding variations in the availability of nutrients in cereal grains is essential to optimize their utilization in the animal diet.

Ensuring optimal concentrations of amino acids (AA) in animal diets is critical to achieve desired growth performance and cost-effectiveness in animal production. Achieving accurate feed formulation, based on digestible AA concentrations, relies on evaluating the AA digestibility of feed ingredients. The AA digestibility of feed ingredients for pigs and broilers is typically evaluated at the terminal ileum to prevent hindgut fermentation [3]. The apparent ileal digestibility (AID) of AA can be determined using the index method by measuring the amounts of dietary AA intake and ileal AA output. Standardized ileal digestibility (SID) values are then calculated by correcting the AID for basal endogenous losses (BEL) of AA. Therefore, SID values have been suggested to provide more predictable estimates for mixed diets in pigs and broilers [4,5].

Determining the energy concentrations of feed ingredients is imperative to minimize feed costs and improve feed efficiency in broilers. Total quantity of energy in feed ingredients can be categorized into gross energy (GE), digestible energy, metabolizable energy (ME), and net energy. The most widely used system for evaluating the energy value of feed ingredients for poultry is through apparent ME (AME) or nitrogen (N)-corrected AME (AMEn) [6]. Two primary collection methods employed for energy evaluation of feed ingredients are the total collection method and the index method [6,7]. Previous studies have highlighted the impact of collection method on ME concentrations and nutrient digestibility of broiler diets [8,9]. However, there is a dearth of comparative studies examining the ME concentrations of cereal grains as determined using the total collection method and the index method.

Therefore, the objectives of the present study were to determine the AME concentrations and ileal AA digestibility of cereal grains and to compare the energy concentrations of cereal grains between the total collection and index methods for 21-day-old broiler chickens.

## MATERIALS AND METHODS

Experimental procedures were reviewed and approved by the Institute for Animal Care and Use Committee of Kyungpook National University, Republic of Korea (approval number: KNU 2022-0177).

### Ingredients and dietary treatments

Five commercially available cereal grains (non-extruded corn, extruded corn, wheat, wheat flour, and barley) were obtained from a local feed supplier in mash form. The energy, chemical, and nutrient compositions of cereal grains are shown in Table 1. The extruded corn was processed using a single-screw extruder (EX620 Matador, Andritz Inc., Graz, Austria) with a die diameter of 12 mm. The extrusion process was performed at 95°C for 50 seconds.

The ileal AA digestibility and AME were determined using the direct method. A N-free diet was prepared to estimate the BEL of AA, as described by Adedokun et al [10]. Five dietary treatments were formulated, each containing one of the cereal grains as the sole source of energy and AA (Table 2). These experimental diets were formulated to meet or exceed the recommended mineral and vitamin levels according to Ross 308 broiler nutrition specifications [11]. Chromium oxide (5 g/kg) was incorporated into all experimental diets as an indigestible index.

### Animals and experimental design

Day-old Ross 308 male broiler chickens were obtained from a local hatchery (Samhwa Breeding, Hongseong, Korea). The birds were initially raised in floor pens (2×2 m) within an environmentally controlled room until they were assigned to their respective dietary treatments. Birds were fed a broiler pre-starter crumble diet (218.2 g/kg crude protein [CP] and 2,998 kcal/kg AMEn) until day 11, followed by a broiler starter crumble diet (192.8 g/kg CP and 2,919 kcal/kg AMEn) from day 11 to 17. On day 17 post-hatch, birds were randomly selected from the floor pens, tagged with identification numbers, and transferred to wire-floored battery cages (60×50×60 cm) within an environmentally controlled room under continuous 24 hours lighting. A total of 336 birds were then allocated to 6 dietary treatments with 8 replicates (7 birds/cage) in a randomized complete block design based on body weight as a blocking factor. The birds were provided experimental diets in mash form for 4 days, the feed and water were offered ad libitum throughout the experiment from day 1 to 21. The room temperature was maintained at 33°C for the first 4 days and gradually decreased by 2°C weekly up to day 21.

### Sample collection

Each period consisted of 2-day adaptation period followed by 2-day excreta collection period. Excreta were collected using the total excreta collection method over 2-day collection period [6]. Waxed paper was placed in trays beneath the cage to collect the excreta, which were gathered twice daily at 0800 and 2000 h. Excreta samples were pooled within each cage, and both the feed intake (FI) and total excreta output were recorded over the 2-day collection period.

On day 21 post-hatch, all birds were euthanized using carbon dioxide asphyxiation. Ileal digesta was collected from the distal two-thirds of the ileum, which constitutes the portion of the small intestine from Meckel’s diverticulum to 1 cm anterior to the ileo-cecal junction. The collected ileal digesta samples were gently rinsed with distilled water and pooled within each cage. The collected excreta and ileal digesta samples were stored in a freezer at −20°C prior to analysis.

### Chemical analysis

The experimental diets were ground using a mill grinder (CT293 Cyclotec; Foss Ltd., Hilleroed, Denmark) through a 1-mm screen for nutrient analysis. Excreta samples were dried in a forced-air drying oven at 55°C and ground to a particle size of less than 1 mm for the analysis. Ileal digesta samples were lyophilized (Vacuum freeze dryer; Samwon Engineering, Busan, Korea) and subsequently ground using a mortar and pestle. The dry matter (DM; method 930.15), CP (method 990.03), ether extract (EE; method 920.39), ash (method 942.05), starch (method 996.11), neutral detergent fiber (method 2002.04), and acid detergent fiber (ADF; method 973.18) contents in the samples were determined. Experimental diets and ileal digesta samples were analyzed for AA content (method 982.30 E [a and b]; [12]). The GE was determined using an adiabatic bomb calorimeter (Parr 6300; Parr Instruments Co., Moline, IL, USA) with benzoic acid serving as a calibration standard. Chromium contents were analyzed according to the method described by Fenton and Fenton [13].

### Calculations

All data were expressed on a DM basis. Energy concentrations of ingredients and test diets were calculated using the following equations:


$$\begin{array}{l} \text{AME of test diet }(\text{kcal}/\text{kg})=(\text{dietary GE intake}-\text{excreta GE output})/\text{FI},\\ \text{AMEs of test diet }(\text{kcal}/\text{kg})=\text{AME}-\text{Fc}\times (0.5\times {\text{N}}_{\text{diet}}-{\text{N}}_{\text{output}})/\text{FI}\times 10^{-3},\\ \text{AMEn of test diet }(\text{kcal}/\text{kg})=\text{AME}-\text{Fc}\times ({\text{N}}_{\text{input}}-{\text{N}}_{\text{output}})/\text{FI}\times 10^{-3},\\ \text{Energy concentration of ingredient }(\text{kcal}/\text{kg})\\ =\text{energy concentration of test diet}\times (100/\text{inclusion rates of cereal gains}) \end{array}$$

where dietary GE intake (kcal), excreta GE output (kcal), and FI (kg) were based on a 2-day collection period; N_input_ (g) represents the amount of N ingested; N_output_ (g) indicates the amount of N voided via excreta; Fc represents the correction factor of 8.22 kcal/g for retained N for birds. The standardized AME (AMEs) value was adjusted for N retention equivalent to a standardized 50% of N intake, corresponding to practical N retention [14]. The AMEn value was calculated by correcting for zero N retention using the factor of 8.22 kcal/g of retained N [15].

The coefficients of AID and SID of AA in experimental diets and BEL of AA in birds were calculated using the following equations [16]:


$$\begin{array}{l} \text{AID}=1-[({\text{Cr}}_{\text{diet}}/{\text{Cr}}_{\text{ileal digesta}})\times ({\text{AA}}_{\text{ileal digesta}}/{\text{AA}}_{\text{diet}})],\\ \text{BEL }(\text{g}/\text{kg of DM intake})=({\text{Cr}}_{\text{diet}}/{\text{Cr}}_{\text{ileal digesta}})\times ({\text{AA}}_{\text{ileal digesta}}),\\ \text{SID}=\text{AID}+(\text{BEL}/{\text{AA}}_{\text{diet}}) \end{array}$$

where Cr_diet_ (%) represents the concentration of chromium in the diet; Cr_ileal digesta_ (%) indicates the concentration of chromium in the ileal digesta; AA_diet_ (%) is the concentration of AA in the diet; AA_ileal digesta_ (%) indicates the concentration of AA in the ileal digesta.

### Statistical analysis

Energy concentrations of cereal grains were analyzed using two-way analysis of variance (ANOVA) with the general linear model procedure of SAS (SAS Inst. Inc., Cary, NC, USA). The source of ingredient, method, and interaction were considered fixed variables. The ileal AA digestibility of cereal grains was analyzed using one-way ANOVA. Treatment means were separated using Tukey’s honestly significant difference test. The experimental unit was a cage, and statistical significance was declared at p<0.05.

## RESULTS

### Dry matter and N retention, and energy concentrations in cereal grains

The DM retention of cereal grains did not exhibit (p>0.05) an interactive effect between the collection method and the source of ingredient (Table 3). The total collection method yielded significantly greater (p<0.05) DM retention of cereal grains compared to the index method. Both non-extruded and extruded corn showed greater DM retention (p<0.05) compared to wheat, wheat flour, and barley. Significant interaction effects (p<0.05) were observed between the collection method and the source of ingredients for N retention, AME-to-GE ratio (AME:GE), AME, AMEs, and AMEn. The N retention, AME:GE, and energy values obtained from the total collection method were greater (p<0.05) than those from the index method. As for the total collection method, N retention, AME:GE, and energy concentrations of both non-extruded and extruded corn were not different and were greater (p<0.05) than those of wheat, wheat flour, and barley. However, with the index method, the N retention of non-extruded corn was the greatest among cereal grains, extruded corn and barley were intermediate, followed by wheat and wheat flour (p<0.05). Additionally, both non-extruded and extruded corn exhibited greater (p<0.05) AME:GE and energy concentrations compared to wheat, wheat flour, and barley, as determined by the index method. Furthermore, the AME and AMEs of barley derived from the index method were lower (p<0.05) compared to wheat and wheat flour.

### Amino acid digestibility in cereal grains

Wheat flour had the greatest AID and SID of N and AA among the cereal grains (Tables 4, 5). The AID and SID of N and AA, except for Arg, Leu, Lys, Met, Ala, and Asp, was greater (p<0.05) in wheat flour compared to non-extruded corn and extruded corn. The AID of AA, except for Ala and Cys, was greater (p<0.05) in the non-extruded corn than in extruded corn. Extruded corn showed comparable (p>0.05) AID of AA to wheat, except for Leu, Ala, Asp, Cys, Glu, and Pro. However, the AID of N and AA in barley, except for Arg, Lys, Thr, Val, and Asp, was lower (p<0.05) compared to the other cereal grains. Non-extruded corn exhibited greater (p<0.05) SID of AA, except for Ala, compared to extruded corn. The SID of AA in extruded corn, except for Arg, Ala, Asp, Glu, and Pro, did not differ (p>0.05) from that in wheat. The SID of N and AA in barley, except for Arg, Lys, Thr, and Asp, was the lowest (p<0.05) among the cereal grains.

## DISCUSSION

The energy and nutrient compositions in non-extruded corn, wheat, and barley in the present study fell within the range of reported values [2,17–20]. However, the reported ranges showed variations in nutrient compositions depending on factors such as growing region, seeding time, and processing conditions [21].

Given that the extruded corn and wheat flour were obtained from a local feed company, any differences between extruded and non-extruded corn, and wheat and wheat flour, may have been due to their source of origin rather than differences in processing methods. Therefore, the present study aims to compare these extruded corn and wheat flours with their conventional counterparts. First, it is important to note that the nutritional compositions in cereal grains may have been altered through the extrusion and milling processes. Extrusion processing involves subjecting grains to consistent cooking under pressure, moisture, and high temperatures, which can improve nutrient digestibility by altering the physicochemical characteristics of grain particles [22]. One notable transformation induced by extrusion is the gelatinization of starch in cereal grains, believed to enhance both nutrient digestibility and palatability [23]. Meanwhile, when proteins in feed ingredients are exposed to heat, the extent of heat damage can be estimated by calculating Lys as a percentage of CP [24]. In the present study, a numerical decrease (from 3.8% to 3.4%) in Lys as a percentage of CP was observed when comparing extruded corn to conventional non-extruded corn from the study of An and Kong [16]. This suggests that the protein in extruded corn may have been damaged during the extrusion process. Thermal degradation can be attributed to both the temperature and duration of extrusion. According to Stein and Bohlke [25], the optimal extrusion temperature ranges from 75°C to 115°C. In the present study, the extrusion temperature was maintained at 95°C, falling within this established range. However, the previous study lacked specific data on extrusion duration. It is plausible that the duration of extrusion in the present study was excessive, potentially leading to increased thermal degradation in the extruded corn. On the other hand, wheat by-products, derived from the industrial process of milling wheat, serve as alternative feedstuffs for animal production and are readily available in Canada and the United States [26]. Unlike wheat by-products, wheat flour is considered essential for human nutrition [27]. Although the higher cost of wheat flour might not make it ideal for animal production, evaluating the nutrient digestibility of wheat flour could prove beneficial if feasible [28]. In the present study, the starch content in wheat flour was numerically greater than that of conventional wheat [2]. The endosperm typically constitutes approximately 80% of a wheat grain’s weight [29]. Wheat co-products such as wheat bran and wheat middlings obtained during the wheat milling process contain relatively low starch concentrations, ranging from 229 to 429 g/kg, compared to wheat. Therefore, the increase in starch content in wheat flour may be partly attributed to the separation of wheat co-products from the endosperm during the milling process.

Energy utilization and concentrations of cereal grains, as determined using the total excreta collection method, were found to be greater than those determined using the index method. Prawirodigdo et al [30] reported that the apparent total tract N digestibility of diets determined using chromium oxide (Cr_2_O_3_) as an indigestible index was lower than that measured through the total fecal collection method for pigs. In a broiler study conducted by Roza et al [8], the ME of pelleted and mash diets, determined by the total collection method, was greater than that determined by the index method using chromium oxide as an indigestible index. Previous studies noted that the decrease in energy values in the index method may be attributed to the low recovery rates of the index compound. The reduced recovery rate of the index can lead to an increase in the indigestibility factor (ratio of chromium concentrations in the diet to chromium concentrations in excreta). The recovery rates of chromium were reported to be 78.9% and 70.5% in growing pigs and Greater rheas [31,32]. In the present study, the recovery of chromium was 69.9%, and this low chromium recovery might primarily stem from issues associated with the index compound. Differences in passage rates between nutrients and the index, uneven distributions due to the electrostatic characteristics of chromium oxide, and analytical errors can contribute to the reduced recovery rate of chromium [8,9]. Moreover, the total collection method for poultry may pose potential issues related to time-based collection, leading to instances where excreta are not collected in trays, and inaccurate excreta collection arises due to losses during tray emptying into containers. These factors may lead to an underestimation of the amount of excreta, thereby overestimating energy utilization and concentrations.

The energy utilization, AME, and AMEn values of non-extruded corn, wheat, and barley in the present study were equal to or exceeded those reported in previous studies [18, 33,34]. Discrepancies in energy values across studies were likely due to variations in feed ingredient cultivars, dietary compositions of experimental diets, duration of excreta collection, and methodological approaches [18]. Wu et al [35] emphasized that the key consideration lies in how the test ingredients should be incorporated into test diets to derive accurate energy values. In the poultry energy system, correcting AME to zero N retention is a widely adopted approach for expressing energy values in feed ingredients [15]. This correction aims to account for variations in energy values of feed ingredients, particularly in cases where experimental diets feature highly imbalanced AA and N contents [7]. Cozannet et al [14] reported that 50% of the N intake by poultry is retained by the body. Consequently, the AMEs value corrects for a representative proportion (50%) of N intake, offering a more practical correction for modern broiler production conditions. In the present study, the AMEs value of cereal grains showed an intermediate value between AME and AMEn. However, wheat and wheat flour exhibited greater AMEs values in contrast to both AME and AMEn, as determined by the index method. This discrepancy observed in the current study was attributed to N retention being less than 50%, potentially leading to a negative N retention equivalent to 50% of the N intake. Therefore, correcting AME to account for N retention resulted in values of 100.8% and 97.5% of the AME of wheat for AMEs and AMEn, respectively. Similarly, correcting AME to N retention resulted in values of 101.0% and 97.2% of the AME of wheat flour for AMEs and AMEn, respectively. As noted by Wise and Adeola [7], AMEs values likely align more closely to test diets that have better balanced AA and N contents.

In the present study, the energy values of extruded corn did not greatly differ from those of conventional non-extruded corn [34]. Despite corn primarily comprising starch, the extrusion process, which involves heat and moisture, is believed to enhance starch digestibility and improve the physical characteristics of feed ingredients [36]. Rodriguez et al [17] reported that extrusion of corn increased ileal starch digestibility, digestible energy, and AME in growing pigs compared to non-extruded corn. The inconsistent results between previous and current studies may arise from variations in energy and starch digestibility of corn. Rodriguez et al [17] observed coefficients of apparent total tract starch and energy digestibility in corn of 0.907 and 0.882, respectively. In contrast, Cervantes-Pahm et al [37] reported greater coefficients of apparent total tract digestibility for starch (0.997) and GE (0.913) in non-extruded corn, indicating its already high digestibility in the small intestine. Hence, the absence of differences between extruded corn and conventional non-extruded corn in this study might be partly attributed to the limited potential for further enhancing starch and GE digestibility to improve the energy values of non-extruded corn. The ileal digestibility of AA of non-extruded corn, wheat, and barley in the present study aligned with reported values [38–41]. Coefficients of AID and SID of AA in extruded corn were numerically lower than those in conventional non-extruded corn [38]. Contrary to the results in the present study, Rodriguez et al [17] noted that extrusion of corn improved the AID and SID of AA compared to non-extruded corn in growing pigs. However, Cho et al [42] observed that a decrease in the true fecal digestibility of indispensable AA, such as Arg, His, Lys, and Val, in extruded corn compared to non-extruded corn. This decrease in the true fecal AA digestibility of extruded corn could be attributed to the thermal degradation of protein occurring during the extrusion process. The protein thermal degradation can result in a reduction in AA concentration in extruded corn, as evidenced in the findings of Cho et al [42]. Consequently, the underestimation of the ileal AA digestibility of feed ingredients may arise from the low dietary AA concentrations, which increase the relative contribution of the BEL of AA to total AA outflows [38].

In the present study, the energy utilization and energy concentrations of wheat flour did not greatly differ from those of conventional wheat [18]. However, the ileal AA digestibility of wheat flour was found to be numerically greater than that of conventional wheat [43]. Fan et al [44] reported that reducing the particle size of wheat linearly increased the apparent total tract digestibility of GE, CP, EE, and ADF, as well as the energy values of wheat. Furthermore, it was anticipated that wheat flour would exhibit greater energy utilization than whole wheat owing to the milling process which separates high fiber by-products from the endosperm. However, contrary to expectations, no notable numerical differences in energy values were observed between wheat flour and conventional wheat in the present study. This may be due to the comparable digestible energy concentrations between wheat by-products, except for millrun and bran [2,26]. Hence, this suggests that the separation of wheat by-products from the endosperm holds more importance than reducing the particle size of wheat in determining nutrient utilization in this case. Regarding the AA digestibility of wheat and wheat flour, wheat by-products separated during the milling process exhibited relatively low ileal AA digestibility compared to whole wheat [2]. Therefore, in this study, the numerically greater ileal AA digestibility of wheat flour compared to conventional wheat may have resulted from the separation of wheat by-products from wheat flour, rather than reducing the particle size of wheat.

In conclusion, the total collection method yields greater energy utilization and concentrations of cereal grains compared to the index method. The AME, AMEn, and AMEs values of both non-extruded and extruded corn were greater than those of wheat, wheat flour, and barley, irrespective of the method used. Further research is warranted to investigate the energy values of high-fiber ingredients and protein sources used in poultry production as determined by different collection methods. The AID and SID of AA in wheat flour were the greatest followed by non-extruded corn, extruded corn, wheat, and barley.

## Figures and Tables

**Table 1:** Energy and nutrient compositions of cereal grains, as-fed basis

Item	Non-extruded corn	Extruded corn	Wheat	Wheat flour	Barley
Dry matter (g/kg)	876.5	885.9	914.4	894.9	918.5
Gross energy (kcal/kg)	4,007	4,059	4,067	4,051	3,939
Crude protein (g/kg)	84.3	79.3	117.9	126	89.3
Ash (g/kg)	13.1	12	18.2	9.9	19.7
Ether extract (g/kg)	36.8	17.1	22.4	29.8	31.5
Neutral detergent fiber (g/kg)	70.8	78.5	179.6	225.0	176.6
Acid detergent fiber (g/kg)	18.4	20.2	26.4	6.0	55.0
Starch (g/kg)	641	643	618	651	555
Indispensable amino acids (g/kg)					
Arginine	4.3	3.9	5.9	5.5	4.5
Histidine	2.4	2.3	2.7	2.8	1.9
Isoleucine	3.1	2.8	3.9	4.1	3.0
Leucine	10.6	9.9	7.7	8.0	6.0
Lysine	3.1	2.7	3.5	3.2	3.5
Methionine	1.8	1.7	1.7	1.9	1.4
Phenylalanine	4.3	4.0	5.2	5.9	4.3
Threonine	3.5	3.2	3.6	3.7	3.2
Valine	4.2	3.9	5.1	5.3	4.4
Dispensable amino acids (g/kg)					
Alanine	6.2	5.9	4.2	4.0	3.7
Aspartic acid	6.4	6.0	6.5	5.6	5.8
Cysteine	2.0	1.9	2.6	2.7	2.1
Glutamic acid	16.6	15.6	33.0	40.5	19.2
Glycine	3.4	3.2	5.0	5.0	3.8
Proline	7.6	7.3	10.7	13.8	8.6
Serine	4.3	4.1	5.4	5.9	3.8
Tyrosine	3.6	3.0	3.5	3.8	2.5

**Table 2:** Ingredient and nutrient compositions of experimental diets fed broilers, as-fed basis

Item	Experimental diets					

	NFD	Non-extruded corn	Extruded corn	Wheat	Wheat flour	Barley
Ingredient compositions (g/kg)						
Corn starch	198.6	-	-	-	-	-
Sucrose	640.0	-	-	-	-	-
Non-extruded corn	-	946.5	-	-	-	-
Extruded corn	-	-	946.5	-	-	-
Wheat	-	-	-	948.6	-	-
Wheat flour	-	-	-	-	948.6	-
Barley	-	-	-	-	-	949.6
Limestone	13.3	12.7	12.7	13.8	14.3	15.1
Dicalcium phosphate	20.6	19.3	19.3	16.1	15.6	13.8
Soybean oil	40.0	-	-	-	-	-
Cellulose	50.0	-	-	-	-	-
Potassium carbonate	3.0	-	-	-	-	-
Sodium bicarbonate	12.0	-	-	-	-	-
Potassium chloride	3.0	-	-	-	-	-
Magnesium oxide	2.0	-	-	-	-	-
Salt	-	4.5	4.5	4.5	4.5	4.5
Vitamin premix^1)^	5.0	5.0	5.0	5.0	5.0	5.0
Mineral premix^2)^	5.0	5.0	5.0	5.0	5.0	5.0
Choline chloride	2.5	2.0	2.0	2.0	2.0	2.0
Chromium oxide	5.0	5.0	5.0	5.0	5.0	5.0
Analyzed composition (g/kg)						
Crude protein	2.3	84.8	80.8	114.5	121.9	86.0
Calculated compositions (g/kg)						
Calcium	8.7	8.7	8.7	8.7	8.7	8.7
Non-phytate phosphorus	4.4	4.4	4.4	4.4	4.4	4.4

NFD, nitrogen-free diet.

1) Vitamin premix supplied per kilogram of diet: retinyl acetate, 24,000 IU; cholecalciferol, 8,000 IU; DL-α-tocopherol acetate, 160 mg/kg; menadione nicotinamide bisulfite, 8 mg/kg; thiamine mononitrate, 8 mg/kg; riboflavin, 20 mg/kg; pyridoxine hydrochloride, 12 mg/kg; D-calcium pantothenate, 40 mg/kg; folic acid, 4 mg/kg; nicotinamide, 12 mg/kg.

2) Mineral premix supplied per kilogram of diet: iron, 120 mg/kg; copper, 320 mg/kg; zinc, 200 mg/kg; manganese, 240 mg/kg; cobalt, 2 mg/kg; selenium, 0.6 mg/kg; iodine, 2.5 mg/kg.

**Table 3:** Coefficient of the dry matter (DM) and nitrogen (N) retention, and energy concentrations of cereal grains fed to 21-day-old broilers determined by the total collection method and index method

Item		DM retention	N retention	AME:GE	AME (kcal/kg DM)	AMEs (kcal/kg DM)	AMEn (kcal/kg DM)
Method	Source						
Total collection	Non-extruded corn	0.911	0.782^a^	0.919^a^	4,201^a^	4,159^a^	4,083^a^
	Extruded corn	0.914	0.786^a^	0.912^a^	4,180^a^	4,139^a^	4,068^a^
	Wheat	0.863	0.723^b^	0.877^b^	3,763^d^	3,728^d^	3,657^d^
	Wheat flour	0.860	0.708^b^	0.871^b^	3,887^bcd^	3,845^cd^	3,750^cd^
	Barley	0.850	0.747^ab^	0.850^b^	3,833^cd^	3,790^cd^	3,684^d^
Index	Non-extruded corn	0.855	0.620^c^	0.878^b^	4,015^b^	3,997^b^	3,926^b^
	Extruded corn	0.845	0.550^d^	0.856^b^	3,921^bc^	3,911^bc^	3,847^bc^
	Wheat	0.777	0.481^e^	0.786^cd^	3,358^f^	3,352^f^	3,285^e^
	Wheat flour	0.786	0.482^e^	0.796^c^	3,481^ef^	3,509^e^	3,395^e^
	Barley	0.763	0.551^d^	0.758^d^	3,503^e^	3,537^e^	3,406^e^
	SEM (n = 8)	0.0079	0.0106	0.0086	37.8	36.8	36.8
Main effect							
Method							
	Total collection	0.880^a^	0.749	0.886	3,973^a^	3,932^a^	3,849^a^
	Index	0.805^b^	0.537	0.815	3,656^b^	3,661^b^	3,572^b^
	SEM (n = 40)	0.0052	0.0059	0.0059	26.1	25.2	25.2
Source							
	Non-extruded corn	0.883^a^	0.701	0.899	4,108^a^	4,078^a^	4,004^a^
	Extruded corn	0.879^a^	0.668	0.884	4,050^a^	4,025^a^	3,957^a^
	Wheat	0.820^b^	0.602	0.832	3,560^c^	3,540^c^	3,471^c^
	Wheat flour	0.823^b^	0.595	0.833	3,684^b^	3,677^b^	3,573^b^
	Barley	0.806^b^	0.649	0.804	3,668^b^	3,663^b^	3,545^bc^
	SEM (n = 16)	0.0064	0.0080	0.0070	31.0	30.1	30.1
p-values							
	Method	<0.001	<0.001	<0.001	<0.001	<0.001	<0.001
	Source	<0.001	<0.001	<0.001	<0.001	<0.001	<0.001
	Method×source	0.129	0.001	0.001	0.002	0.005	0.003

AME, apparent metabolizable energy; GE, gross energy; AMEs, standardized apparent metabolizable energy; AMEn, nitrogen-corrected apparent metabolizable energy; SEM, standard error of the mean.

a–f Least squares means within a column without a common superscript differ (p<0.05).

**Table 4:** Coefficient of apparent ileal digestibility of nitrogen and amino acids of cereal grains fed to 21-day-old broilers

Item	Non-extruded corn	Extruded corn	Wheat	Wheat flour	Barley	RMSE	p-value
n	8	8	8	7	8		
Nitrogen	0.855^b^	0.825^b^	0.838^b^	0.924^a^	0.752^c^	0.0224	<0.001
Indispensable amino acids							
Arginine	0.906^a^	0.854^b^	0.814^bc^	0.947^a^	0.793^c^	0.0282	<0.001
Histidine	0.885^b^	0.846^c^	0.834^c^	0.938^a^	0.781^d^	0.0231	<0.001
Isoleucine	0.877^b^	0.817^c^	0.856^bc^	0.941^a^	0.772^d^	0.0276	<0.001
Leucine	0.925^a^	0.895^b^	0.866^c^	0.946^a^	0.800^d^	0.0188	<0.001
Lysine	0.848^a^	0.762^b^	0.775^b^	0.892^a^	0.755^b^	0.0406	<0.001
Methionine	0.930^a^	0.879^b^	0.877^b^	0.945^a^	0.800^c^	0.0227	<0.001
Phenylalanine	0.900^b^	0.862^c^	0.877^bc^	0.955^a^	0.793^d^	0.0200	<0.001
Threonine	0.782^b^	0.693^cd^	0.725^c^	0.839^a^	0.670^d^	0.0330	<0.001
Valine	0.803^b^	0.738^cd^	0.763^bc^	0.886^a^	0.704^d^	0.0305	<0.001
Dispensable amino acids							
Alanine	0.913^a^	0.878^a^	0.792^b^	0.906^a^	0.750^c^	0.0255	<0.001
Aspartic acid	0.858^a^	0.813^b^	0.771^c^	0.880^a^	0.737^c^	0.0285	<0.001
Cysteine	0.842^bc^	0.816^c^	0.851^b^	0.905^a^	0.787^d^	0.0189	<0.001
Glutamic acid	0.924^b^	0.895^c^	0.935^b^	0.979^a^	0.848^d^	0.0147	<0.001
Glycine	0.820^b^	0.772^c^	0.795^bc^	0.921^a^	0.729^d^	0.0274	<0.001
Proline	0.891^c^	0.865^d^	0.914^b^	0.966^a^	0.828^e^	0.0145	<0.001
Serine	0.847^b^	0.799^c^	0.817^bc^	0.913^a^	0.731^d^	0.0230	<0.001
Tyrosine	0.888^b^	0.857	0.849^c^	0.927^a^	0.785^d^	0.0202	<0.001

RMSE, root mean square error.

a–e Least squares means within a row without a common superscript differ (p<0.05).

**Table 5:** Coefficient of standardized ileal digestibility of nitrogen and amino acids (AA) of cereal grains fed to 21-day-old broilers^1)^

Item	Non-extruded corn	Extruded corn	Wheat	Wheat flour	Barley	RMSE	p-value
n	8	8	8	7	8		
Nitrogen	0.896^b^	0.869^b^	0.870^b^	0.949^a^	0.794^c^	0.0193	<0.001
Indispensable amino acids							
Arginine	0.934^a^	0.885^b^	0.834^c^	0.964^a^	0.819^c^	0.0255	<0.001
Histidine	0.908^b^	0.871^c^	0.854^c^	0.954^a^	0.809^d^	0.0217	<0.001
Isoleucine	0.912^b^	0.856^c^	0.882^bc^	0.961^a^	0.807^d^	0.0236	<0.001
Leucine	0.942^a^	0.913^b^	0.889^b^	0.963^a^	0.829^c^	0.0169	<0.001
Lysine	0.882^a^	0.804^b^	0.807^b^	0.919^a^	0.786^b^	0.0364	<0.001
Methionine	0.949^a^	0.903^b^	0.897^b^	0.962^a^	0.827^c^	0.0211	<0.001
Phenylalanine	0.926^b^	0.890^c^	0.898^c^	0.971^a^	0.819^d^	0.0179	<0.001
Threonine	0.848^b^	0.773^cd^	0.793^c^	0.897^a^	0.746^d^	0.0293	<0.001
Valine	0.865^b^	0.808^c^	0.814^c^	0.928^a^	0.763^d^	0.0288	<0.001
Dispensable amino acids							
Alanine	0.934^a^	0.900^a^	0.823^b^	0.932^a^	0.784^c^	0.0239	<0.001
Aspartic acid	0.897^a^	0.857^b^	0.812^c^	0.918^a^	0.780^c^	0.0253	<0.001
Cysteine	0.900^b^	0.873^c^	0.894^bc^	0.944^a^	0.842^d^	0.0176	<0.001
Glutamic acid	0.943^b^	0.916^c^	0.944^b^	0.986^a^	0.864^d^	0.0130	<0.001
Glycine	0.864^b^	0.820^c^	0.826^c^	0.946^a^	0.769^d^	0.0253	<0.001
Proline	0.917^b^	0.891^c^	0.932^b^	0.978^a^	0.849^d^	0.0138	<0.001
Serine	0.895^b^	0.854^c^	0.859^c^	0.946^a^	0.787^d^	0.0209	<0.001
Tyrosine	0.919^b^	0.890^c^	0.880^c^	0.952^a^	0.825^d^	0.0177	<0.001

RMSE, root mean square error.

1) Coefficients of standardized ileal digestibility were calculated by correcting coefficients of apparent ileal digestibility for basal endogenous losses of nitrogen or amino acids. Basal endogenous losses (mg/kg of dry matter intake) were determined as: nitrogen, 3,962; arginine, 113; histidine, 51; isoleucine, 91; leucine, 175; lysine, 110; methionine, 33; phenylalanine, 108; threonine, 233; valine, 224; alanine, 130; aspartic acid, 248; cysteine, 146; glutamic acid, 306; glycine, 148; proline, 195; serine, 204; and tyrosine, 108.

a–d Least squares means within a row without a common superscript differ (p<0.05).
